# Effects of Body Mass Index on Risks for Ischemic Stroke, Thromboembolism, and Mortality in Chinese Atrial Fibrillation Patients: A Single-Center Experience

**DOI:** 10.1371/journal.pone.0123516

**Published:** 2015-04-07

**Authors:** Hai-Jun Wang, Quan-Jin Si, Zhao-Liang Shan, Yu-Tao Guo, Kun Lin, Xiao-Ning Zhao, Yu-Tang Wang

**Affiliations:** 1 Department of Geriatric Cardiology, Chinese PLA General Hospital, Beijing, China; 2 Healthcare Department 2, Hainan Branch of Chinese PLA General Hospital, Sanya, China; 3 Department of Cardiology, Chinese PLA General Hospital, Beijing, China; University of Texas Health Science Center at San Antonio, UNITED STATES

## Abstract

**Background:**

Obesity is considered to be related to recurrence of atrial fibrillation (AF), left atrial thrombus formation, and atrial remodeling. However, whether obesity is an independent risk factor for stroke and other thromboembolic events is still controversial.

**Objective:**

This study aimed to investigate the effects of body mass index (BMI) on the risks of stroke, thromboembolism, and mortality in AF patients.

**Methods:**

Patients who were diagnosed with nonvalvular AF were included in this observational, retrospective study. The study population was stratified by BMI at baseline. The Cox proportional hazard model was adopted to calculate adjusted hazard ratios of risk factors for adverse clinical events (stroke, thromboembolism, and mortality).

**Results:**

A total of 1286 AF patients (males, 78.30%; mean age, 74.50 years; 94.48% paroxysmal AF) were followed up for a median of 2.1 years (IQR: 1.5–2.9 years). Overall, 159 patients died. A total of 84 strokes and 35 thromboembolic events occurred. Multivariate analysis showed that overweight (25.0≤BMI<30.0 kg/m^2^) and age ≥75 years were independent risk factors for ischemic stroke (both P<0.01). Obesity (BMI ≥30.0 kg/m^2^), age ≥75 years, persistent/permanent AF, and prior thromboembolism were independent risk factors for thromboembolism (all P<0.05). Underweight (BMI <18.5 kg/m^2^), age ≥75 years, prior ischemic stroke/transient ischemic attack, renal dysfunction, and heart failure were independent risk factors for all-cause deaths (all P<0.05).

**Conclusions:**

Overweight or obesity may be a risk factor of ischemic stroke and thromboembolism in AF patients. Excessive low weight is significantly associated with increased all-cause mortality.

## Introduction

Atrial fibrillation (AF) is the most common type of rhythm disorder. AF is responsible for the majority of ischemic strokes and thromboembolism, and is also closely related to all-cause mortality [1]. The current risk stratification schemes for ischemic stroke in patients with nonvalvular AF vary widely and have only limited predictive effects.

Obesity is considered to be one of the risk factors for hypertension, coronary heart disease, diabetes mellitus, and congestive heart failure. In recent years, obesity has been repeatedly established as an independent predictor of incident AF and higher overall mortality [2]. Some studies [3,4] have shown that obesity is associated with progression of AF, inflammation, and hypofibrinolysis, suggesting that obesity might be a risk factor for thromboembolic events. Other studies [5–7] have also indicated that obesity can independently predict the occurrence of AF, left atrial thrombus formation, and atrial structural remodeling. However, widely applied recommendations, such as the CHADS_2_ (congestive heart failure, hypertension, age ≥75, diabetes mellitus, and prior stroke or transient ischemic attack) score [8] or the CHA_2_DS_2_-VASc (congestive heart failure, hypertension, age ≥75 years, diabetes mellitus, stroke, vascular disease, age 65–74 years, sex category) score [9], consider obesity as an accompanying risk factor for stroke with AF. Thus far, the prognostic impact of obesity on outcomes among AF patients has not been well investigated.

Therefore, whether obesity is an independent risk factor for ischemic stroke and thromboembolism is still controversial [10,11]. This study aimed to investigate the effects of BMI on the risks of stroke and thromboembolism in patients with nonvalvular AF.

## Methods

### 1 Study population

We retrospectively reviewed the data of a total of 1286 hospitalized patients with AF who were admitted in Chinese PLA General Hospital (one of the largest general hospitals, with over 4000 beds, in Beijing, China) from January 2008 to June 2010. All of the patients have a permanent and personal registration number in the hospital, which allows complete collection of patients’ outpatient and inpatient attendance. The hospital electronic medical database records all of the medical histories, therapeutic procedures, thromboembolic events, deaths, and laboratory and imaging data. Information on comorbidities and events were based on the International Classification of Disease, 10th Revision (ICD-10) codes 427.3 and 427.31/I48. The inclusion criteria included a history of paroxysmal, persistent, or permanent AF. Patients with valvular heart diseases, those who had been treated with radiofrequency catheter ablation, and those in the acute phase of cardiovascular diseases or uncontrolled infection were all excluded.

### 2 Methods

General information, including height (m) and body weight (kg), was recorded on admission. Body weight was measured by a digital scale with patients wearing either light clothing or underwear, and was recorded to the nearest 100 g. BMI was calculated as weight (kg)/ [height (m)]^2^. The patients were divided into four groups according to the criteria stratified by World Health Organization guidelines [12]: underweight group (BMI<18.5 kg/m^2^), normal weight group (18.5≤BMI<25.0 kg/m^2^), overweight group (25.0≤BMI<30.0 kg/m^2^), and obesity group (BMI≥30.0 kg/m^2^). The risk of stroke was assessed by the CHADS_2_ risk score and CHA_2_DS_2_-VASc risk score. Patients were followed up using electronic database and telephone calls to record major clinical adverse events, including ischemic stroke, thromboembolism, and all-cause deaths. Paroxysmal AF was defined as lasting less than 7 days with spontaneous termination according to published guidelines. Persistent AF and permanent AF were considered as non-paroxysmal AF. Ischemic stroke was defined as sudden onset of neurological deficit lasting >24 h and was confirmed by computed tomography (CT) or magnetic resonance imaging (MRI). Thromboembolism was defined as sudden occlusion of an artery to a visceral organ or extremity documented by imaging or pathology and not attributable to concomitant atherosclerosis or other etiologies. Renal dysfunction was defined as the presence of chronic dialysis or an estimated glomerular filtration rate (eGFR) <60 mL/min per 1.73 m^2^, using the abbreviated equation developed by the Modification of Diet in Renal Disease study: eGFR = 186×[SCR]^−1.154^×[age]^−0.203^×[0.742 if female]×1.233, where SCR is serum creatinine and 1.233 is the adjustment coefficient for Chinese [13]. Information of the endpoints was collected from the medical records and questionnaires that were answered by patients themselves or relatives.

### 3 Ethics statement

The written informed consents were obtained from all subjects or their designated relatives for their clinical records to be used in this study. The Institutional Review Board of the PLA General Hospital approved this retrospective study.

### 4 Statistical analysis

All statistical analyses were performed with IBM SPSS statistics version 19.0 (SPSS, Inc., Chicago, IL). Continuous variables are shown as mean ± SD. Data with a non-normal distribution are shown as median with interquartile range (IQR). Univariate analysis was computed using the un-paired independent samples t test for continuous variables and chi-square test or Fisher’s exact test (when the 2×2 table had <5 patients) for categorical variables. The Cox proportional hazard model was adopted to calculate the adjusted hazard ratio (HR) of risk factors for clinical adverse events. Confidence intervals (CIs) were 95%. A two-sided P value <0.05 was considered significant.

## Results

### 1 Patients’ characteristics

A total of 1286 patients with AF (males, 78.30%; mean age at baseline, 74.5±13.86 years) were followed up for a median of 2.1 years (IQR: 1.5–2.9 years). In this cohort, 81 patients were at low risk (CHA_2_DS_2_-VASc risk score = 0) and 19 of them received anti-platelet drugs. A total of 147 (11.43%) patients were at intermediate risk (CHA_2_DS_2_-VASc risk score = 1), 85 of whom received aspirin or clopidogrel. Only 171 patients (13.30%) received oral anticoagulation therapy, despite 1058 patients (82.27%) having guideline recommendations (CHA_2_DS_2_-VASc risk score ≥2) [9].

Clinical characteristics of the study population and an overall analysis comparing all of the BMI groups are shown in Table 1. In this cohort, 44 (3.42%) patients met the BMI criteria for underweight, 482 (37.48%) met the BMI criteria for overweight, and 92 (7.15%) met the BMI criteria for obesity. Obese patients were relatively younger (P<0.01) and had a higher rate of hypertension and current smoking, and a lower rate of prior stroke and coronary artery disease than did those with normal weight (P<0.05). Overweight patients had a higher rate of hypertension and a lower rate of heart failure and prior stroke than did those with normal weight (all P<0.05). Diastolic blood pressure was higher in obese patients and overweight patients than in those with normal weight (both P<0.01). Overweight patients and obese patients were treated more frequently with oral anticoagulation therapies than normal weight patients, respectively (P<0.05). The highest proportion of patients over 75 years old was 81.82% in underweight patients. No significant differences in complications were observed among different BMI groups.

**Table 1:** Baseline characteristics of Chinese patients with atrial fibrillation according to body mass index.

		Body mass index				P value		
Variables	Entire cohort (n = 1286)	Underweight (<18.5 kg/m^2^, n = 44)	Normal weight (18.5≤BMI<25.0 kg/m^2^, n = 668)	Overweight (25.0≤BMI<30.0 kg/m^2^, n = 482)	Obesity (≥30.0 kg/m^2^, n = 92)	Underweight vs normal weight	Overweight vs normal weight	Obesity vs normal weight
**Demographic profile**								
Age (y), mean (SD)	74.50(13.86)	82.08(9.49)	76.59(13.35)	72.15(13.88)	67.92(14.60)	0.009	<0.001	<0.001
<65 y, n (%)	295(22.94)	3(6.82)	112(16.77)	143(29.67)	37(40.22)	0.082	<0.001	<0.001
65 to <75 y, n (%)	235(18.27)	5(11.36)	117(17.51)	94(19.50)	19(20.65)	0.294	0.390	0.462
≥75 y, n (%)	756(58.79)	36(81.82)	439(65.72)	245(50.83)	36(39.13)	0.028	<0.001	<0.001
Sex (males), n (%)	1007(78.30)	34(77.27)	520(77.84)	390(80.91)	63(68.48)	0.930	0.206	0.046
BMI (kg/m^2^), mean (SD)	24.64(3.59)	17.19(1.37)	22.49(1.67)	26.85(1.37)	32.22(2.38)	<0.001	<0.001	<0.001
Current smoking, n (%)	140(10.89)	4(9.09)	63(9.43)	57(11.83)	16(17.39)	0.940	0.190	0.019
SBP (mmHg), mean (SD)	130.21(18.01)	127.48(21.28)	129.49(18.33)	131.29(17.61)	130.04(15.78)	0.486	0.094	0.437
DBP (mmHg), mean (SD)	72.48(11.87)	68.07(11.40)	71.20(11.80)	74.09(11.51)	75.42(12.81)	0.088	<0.001	0.001
**AF-specific profile**								
AF duration (y), mean (SD)	7.52(9.22)	6.11(8.54)	8.06(9.41)	7.10(9.25)	6.39(7.78)	0.174	0.083	0.104
Paroxysmal AF, n (%)	1215(94.48)	40(90.91)	627(93.86)	462(95.85)	86(93.48)	0.436	0.137	0.886
CHADS_2_ score, mean (SD)	2.36(1.53)	2.20(1.39)	2.50(1.54)	2.22(1.50)	2.14(1.55)	0.209	0.002	0.036
CHA_2_DS_2_VASc score, mean (SD)	3.62(1.90)	3.55(1.72)	3.84(1.87)	3.40(1.93)	3.20(1.90)	0.317	<0.001	0.002
**Morbidity profile**								
Heart failure, n (%)	263(20.45)	8(18.18)	154(23.05)	81(16.80)	20(21.74)	0.455	0.010	0.778
Hypertension, n (%)	820(63.76)	18(40.91)	406(60.78)	329(68.26)	67(72.83)	0.009	0.009	0.025
Diabetes mellitus, n (%)	387(30.09)	7(15.91)	199(29.79)	154(31.95)	27(29.35)	0.049	0.433	0.931
Stroke/TIA, n (%)	295(22.94)	13(29.55)	173(25.90)	94(19.50)	15(16.30)	0.594	0.011	0.046
Cerebral bleeding, n (%)	61(4.74)	1(2.27)	32(4.79)	21(4.36)	7(7.61)	0.442	0.729	0.251
PVD, n (%)	227(17.65)	4(9.09)	128 (19.16)	83(17.22)	12(13.04)	0.096	0.401	0.156
CAD, n (%)	822(63.92)	28(63.64)	440(65.87)	303(62.86)	51(55.43)	0.763	0.293	0.050
Revascularization, n (%)	157(12.21)	0(0)	75(11.23)	68(14.11)	14(15.22)	0.019	0.144	0.265
Prior TE, n (%)	57(4.43)	1(2.27)	33(4.94)	20(4.15)	3(3.26)	0.422	0.528	0.477
Renal dysfunction, n (%)	126(9.80)	5(11.36)	70(10.48)	43(8.92)	8(8.70)	0.853	0.381	0.597
Malignancy, n (%)	191(14.85)	15(34.09)	98(14.67)	70(14.52)	8(8.70)	<0.001	0.944	0.121
**Procedure profile**								
Warfarin therapy, n (%)	171(13.30)	3(6.82)	72(10.78)	74(15.35)	22(23.91)	0.407	0.033	<0.001
Anti-platelet drugs, n (%)	939(73.02)	32(72.73)	489(73.20)	358(74.27)	60(65.22)	0.945	0.684	0.109
Follow-up (y), mean (SD)	2.23(0.89)	2.07(0.85)	2.31(0.91)	2.16(0.89)	2.13(0.70)	0.089	0.007	0.076

Data are shown as mean (SD) or n (%).

BMI: body mass index; AF: atrial fibrillation; TIA: transient ischemic attack; SBP: systolic blood pressure; DBP: diastolic blood pressure; PVD: peripheral vascular disease; CAD: coronary artery disease; TE: thromboembolism. CHADS_2_ score: assigning one point each for congestive heart failure, hypertension, age ≥75 and diabetes mellitus, and two points for a prior stroke or transient ischemic attack. CHA2DS2-VASc score: assigning one point each for congestive heart failure, hypertension, diabetes mellitus, vascular disease, age 65–74 years and sex category, and two points each for age ≥75 years and prior stroke.

### 2 Clinical events

**Fig 1:**
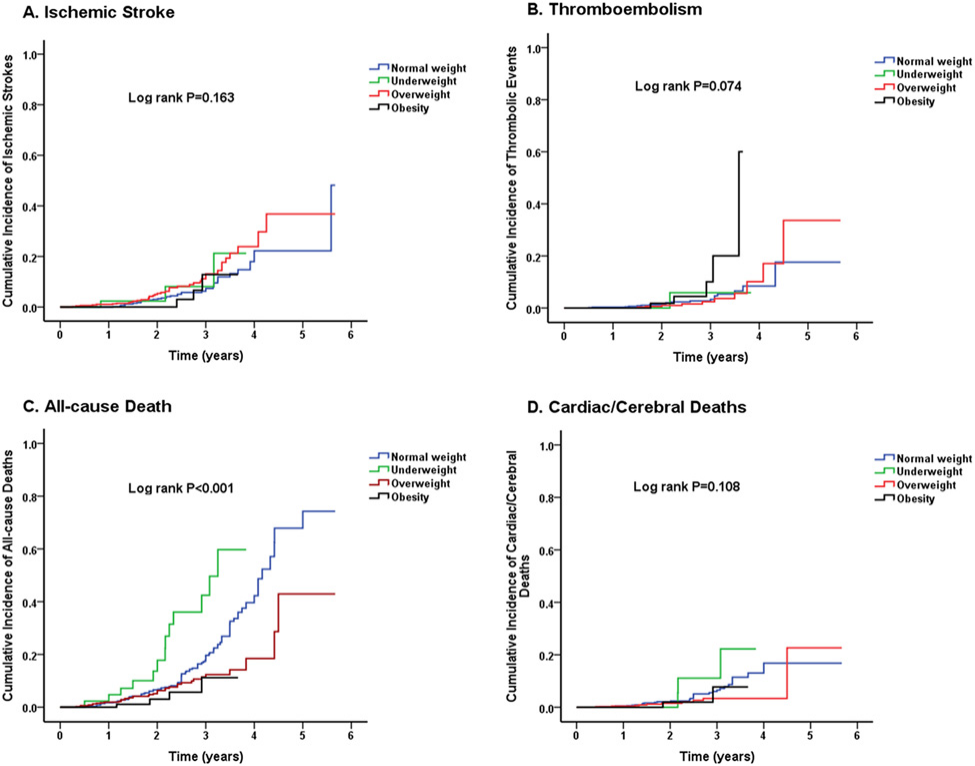
Kaplan-Meier estimates of cumulative incidences of (A) ischemic stroke, (B) thromboembolism, (C) all-cause death and (D) cardiac/cerebral death.

Clinical outcomes and crude incidence rates of patients with AF according to BMI is listed in Table 2. During the 2869 person-years of follow-up (median: 2.1 years), 159 (12.36%) patients died, 33 (2.57%) died of cardiac death, and 14 (1.09%) died of cerebral death. A total of 84 (6.53%) patients had stroke, 35 (2.72%) had other thromboembolic events, and 35 (2.72%) had myocardial infarction. The annual all-cause mortality in underweight patients was approximately 14.29%, and declined stepwise to 6.75%, 3.64% and 2.04% in normal weight, overweight and obesity patients, respectively (overall P<0.01). Moreover, the annual events of cardiac/ cerebral death and myocardial infarction increased from the BMI categories of obesity to underweight. The annual events of thromboembolism were highest in obese patient (2.55%), intermediate in underweight and normal weight patients (1.10% and 1.23%), and lowest in overweight patients (0.96%). The prevalence of stroke was higher in overweight patients (3.74%) than that in normal weight patients (2.53%). On univariate analysis, the cumulative incidence of all-cause mortality was highest in underweight subjects, followed by normal weight, overweight and obese subjects. The cumulative incidence of ischemic stroke was highest in overweight subjects and that of thromboembolism was highest in obese patients (Fig 1).

**Table 2:** Adverse event rates for 1286 patients with atrial fibrillation according to categories of body mass index.

Adverse events	Underweight (BMI<18.5 kg/m^2^)		Normal weight (18.5≤BMI<25.0 kg/m^2^)		Overweight (25.0≤BMI<30.0 kg/m^2^)		Obesity(BMI≥30.0 kg/m^2^)		P value
	No. of events, n (%)	Incidence rate, (%/yr)	No. of events, n (%)	Incidence rate, (%/yr)	No. of events, n (%)	Incidence rate, (%/yr)	No. of events, n (%)	Incidence rate, (%/yr)	
Stroke	3(6.82)	3.30	39(5.84)	2.53	39(8.09)	3.74	3(3.26)	1.53	0.255
TE	1(2.27)	1.10	19(2.84)	1.23	10(2.07)	0.96	5(5.43)	2.55	0.335
MI	2(4.55)	2.20	27(4.04)	1.75	6(1.24)	0.58	0(0)	0	0.245
All-cause death	13(29.55)	14.29	104(15.57)	6.75	38(7.88)	3.64	4(4.35)	2.04	<0.001
Cardiac/cerebral death	3(6.82)	3.30	32(4.79)	2.08	10(2.07)	0.96	2(2.17)	1.02	0.053
Cardiac death	2(4.55)	2.20	25(3.74)	1.62	4(0.83)	0.38	2(2.17)	1.02	0.017
Cerebral death	1(2.27)	1.10	7(1.05)	0.45	6(1.24)	0.58	0(0)	0	0.636

BMI: body mass index; TE: thromboembolism; MI: myocardial infarction.

### 3 Multivariate analyses

HRs using normal weight patients as the reference after the end of the follow-up period are shown in Table 3. At the end of the follow-up, the crude HRs for ischemic stroke in the overweight patients and thromboembolism in the obese patients were higher than those in the normal weight group, and remained significant (HR: 1.82, 95% CI: 1.16–2.84, P = 0.009; and HR: 4.83, 95% CI: 1.75–13.36, P = 0.002, respectively) after adjusting for risk factors included in the CHADS2-VAS risk score and for other risk factors. The HR in the underweight group was higher than that in the normal weight group for all-cause death (HR: 2.01, 95% CI: 1.12–3.60, P = 0.019) after adjusting for other risk factors. The HR in the overweight patients was lower than that in the normal weight patients for all-cause death (HR: 0.68, 95%CI: 0.47–0.99, P = 0.044). There were no significant differences in HRs for cardiac/cerebral death among the different BMI categories. Additionally, after adjusting for other risk factors, age ≥75 years was an independent risk factor for ischemic stroke, thromboembolism, and all-cause death (HR: 3.12, 95% CI: 1.76–5.53, P<0.001; HR: 5.18, 95% CI: 1.53–17.55, P = 0.008; and HR: 2.58, 95% CI: 1.61–4.12, P<0.001, respectively). Persistent AF and permanent AF were more closely related to thromboembolism than paroxysmal AF (HR: 2.81, 95% CI: 1.25–6.32, P = 0.012). A history of prior thromboembolism was an independent risk factor for recurrence or other new onset thromboembolic events (HR: 7.81, 95% CI: 3.66–16.69, P<0.001). Heart failure, prior stroke/transient ischemic attack, and renal dysfunction were independent risk factors for all-cause death (HR: 1.58, 95%CI: 1.12–2.23, P = 0.009; HR1.71, 95%CI: 1.23–2.36, P<0.001; and HR: 2.61, 95%CI: 1.80–3.78, P<0.001) and cardiac/cerebral death (HR: 2.49, 95%CI: 1.31–4.73, P = 0.006; HR: 2.11, 95%CI: 1.12–3.99, P = 0.021; and HR: 2.66, 95%CI: 1.25–5.65, P = 0.011, respectively) (Table 3).

**Table 3:** Hazard ratios (95% CI) for ischemic stroke, thromboembolism, all-cause death and cardiac/cerebral death among 1286 patients at the end of the follow-up period.

Entire cohort	Univariate model			Multivariate model		
	HR	95% CI	P value	HR	95% CI	P value
**Ischemic stroke**						
BMI category (kg/m^2^)						
Underweight (BMI <18.5)	1.54	0.48–4.99	0.472	1.29	0.40–4.20	0.668
Normal weight (18.5≤BMI<25.0)	1.00 (reference)			1.00 (reference)		
Overweight (25.0≤BMI <30.0)	1.59	1.02–2.49	0.040	1.82	1.16–2.84	0.009
Obesity (BMI ≥30.0)	0.78	0.24–2.55	0.685	1.02	0.31–3.34	0.969
Age ≥ 75 y	2.90	1.65–5.10	<0.001	3.12	1.76–5.53	<0.001
**TE**						
BMI category (kg/m^2^)						
Underweight (BMI <18.5)	1.15	0.15–8.61	0.893	1.01	0.13–7.65	0.996
Normal weight (18.5≤BMI<25.0)	1.00 (reference)			1.00 (reference)		
Overweight (25.0≤BMI <30.0)	0.84	0.39–1.80	0.496	0.81	0.37–1.78	0.594
Obesity (BMI ≥30.0)	3.15	1.15–8.62	0.025	4.83	1.75–13.36	0.002
Age ≥ 75 y	6.12	1.87–20.11	0.003	5.18	1.53–17.55	0.008
Non-paroxysmal AF	4.38	2.00–9.42	<0.001	2.81	1.25–6.32	0.012
Prior TE	9.76	4.66–20.45	<0.001	7.81	3.66–16.69	<0.001
**All-cause death**						
BMI category (kg/m^2^)						
Underweight (BMI<18.5)	2.51	1.40–4.47	0.002	2.01	1.12–3.60	0.019
Normal weight (18.5≤BMI<25.0)	1.00 (reference)			1.00 (reference)		
Overweight (25.0≤BMI <30.0)	0.57	0.39–0.83	0.003	0.68	0.47–0.99	0.044
Obesity (BMI ≥30.0)	0.39	0.14–1.05	0.062	0.51	0.19–1.39	0.189
HF	2.17	1.55–3.04	<0.001	1.58	1.12–2.23	0.009
Prior stroke/TIA	2.11	1.54–2.91	<0.001	1.71	1.23–2.36	0.001
Renal dysfunction	2.93	2.03–4.22	<0.001	2.61	1.80–3.78	<0.001
Age ≥ 75 y	3.82	2.43–6.00	<0.001	2.58	1.61–4.12	<0.001
**Cardiac/cerebral death**						
BMI category (kg/m^2^)						
Underweight (BMI<18.5)	1.83	0.56–5.99	0.318	1.55	0.47–5.10	0.336
Normal weight (18.5≤BMI<25.0)	1.00 (reference)			1.00 (reference)		
Overweight (25.0≤BMI <30.0)	0.49	0.24–1.01	0.052	0.54	0.26–1.12	0.098
Obesity (BMI ≥30.0)	0.61	0.15–2.55	0.497	0.66	0.15–2.90	0.582
HF	2.53	1.38–4.65	0.003	2.49	1.31–4.73	0.006
Prior stroke/TIA	2.53	1.42–4.50	0.002	2.11	1.12–3.99	0.021
Renal dysfunction	2.57	1.28–5.16	0.008	2.66	1.25–5.65	0.011

Univariate model: Univariable analysis of adverse events using a Cox proportional hazards model.

Multivariate model: Cox proportional hazards model adjusted for congestive heart failure, hypertension, diabetes mellitus, prior stroke/TIA, peripheral vascular disease, previous TE other than stroke/TIA, age ≥75 years, smoking, paroxysmal AF, renal dysfunction, anticoagulation therapy, and sex category.

BMI: body mass index; HF: heart failure; TIA: transient ischemic attack; TE: thromboembolism.

## Discussion

Thromboembolic complications are adverse outcomes in patients with AF. Additionally, thromboembolic events have a similar rate in paroxysmal and persistent AF [14]. As is known, BMI is now commonly used to estimate body composition and identify overweight and obese patients. However, whether an increased BMI in AF patients can increase the risks for thromboembolic events is unknown. This study investigated 1286 nonvalvular AF patients, and showed that an increased BMI category was potentially related to a higher risk of ischemic stroke and thromboembolism. Similarly, being underweight could predict all-cause death independently of those clinical variables in the CHA_2_DS_2_-VASc risk score. Because obesity is an increasing problem at present, our results might have important implications for the management of AF.

Current risk stratification schemes for thromboembolism in AF have only limited predictive effects and additional prognostic variables are still being sought [15,16]. Obesity is often, but not always, considered to be a component of metabolic syndrome, a condition that has a definite thromboembolic risk. The effect of obesity on AF outcomes has not been well investigated. Therefore, the association between obesity and thromboembolism in AF patients is unclear. A cross-sectional study by Novo *et al*. [10] found no association between obesity and the risk of thromboembolic events in a study of 480 AF patients. However, other studies have supported the notion that obesity is associated with a worse prognosis, such as stroke and thromboembolism in AF patients. In our study, we found that the risk ratios for ischemic stroke and thromboembolism in overweight and obese patients were higher than those in normal weight patients, even after adjusting for CHA_2_DS_2_-VASc risk factors. The HR for all-cause death in the underweight group was higher than that in the normal weight group after adjusting for other risk factors. There was no significant difference in the risk ratio for cardiac/cerebral death among the different BMI categories. The results of this study are partly in accordance with other studies on AF patients [17]. Overvad *et al*. demonstrated overweight and obesity were risk factors for stroke, thromboembolism, or death (a composite endpoint) in their large cohort of AF patients. Previous studies have suggested that obesity in AF patients is associated with an increased risk of progression of AF, a higher rate of AF [18], an increased rate of recurrence of atrial fibrillation [19], and a higher prevalence of a left atrial/left atrial appendage thrombus formation [6]. Additionally, obesity is considered as an independent predictor for procedural failure after catheter ablation [20] in either paroxysmal or persistent/permanent AF [21]. The mechanisms of the higher risk for ischemic stroke and thromboembolism among overweight and obese patients may partly be due to unmeasured comorbidity not encompassed in the CHADS_2_ and CHA_2_DS_2_-VASc risk scores. Obese patients often have an unhealthy lifestyle, obstructive sleep apnea, insulin resistance, and sympathetic nervous system activation status, which are also related to thrombotic adverse events [22]. Obese patients are also often associated with a “pro-thrombotic state,” meaning a higher presence of thrombotic cofactors [23]. Our study showed that overweight and obesity in AF patients were risk factors of thrombosis and stroke. However, further studies are necessary to determine whether weight reduction up to the normal range decreases the risk of AF, and hence stroke and thromboembolism.

In our study, the participants were followed in a hospital follow-up center with a limited loss to follow-up. Therefore, our study was not likely subject to selection bias. The proportion of elderly patients with AF receiving warfarin therapy was low, mainly because antiplatelet therapy was administered to some high-risk patients who should have been treated with anticoagulation in clinical practice. The causes of lack use of warfarin may include poor compliance of elderly patients, a high bleeding risk associated with warfarin, frequent and complex means of monitoring the international normalized ratio, and incorrect understanding of warfarin equivalent to aspirin or clopidogrel. Therefore, anticoagulation therapy is a challenge for older patients with AF in China. In addition, the lack use of warfarin in this study might affect the incidences of cardiovascular events, which must be taken into account when administering warfarin in order to minimize the risk of under-anticoagulation or over-anticoagulation.

After a mean follow-up of 2.1 years, we found that underweight patients were associated with a significantly higher risk of all-cause mortality, overweight patients were associated with a higher risk of ischemic stroke, and obese patients were associated with a higher risk of thromboembolism compared with normal weight patients, after adjusting for risk factors included in the CHA_2_DS_2_-VASc risk score and for other risk factors. We did not find that obese patients had worse survival as compared with normal-weight patients. These findings suggest that there might be an obesity paradox among AF patients [24]. Some previous studies [25] support our findings. A possible explanation for the obesity paradox is the increased use of standard medical therapies such as angiotensin receptor blockers/angiotensin-converting enzyme inhibitors and statins in obese patients. Protection against malnutrition and energy wastage by obesity is another possible explanation [25]. Obese patients may have more metabolic reserve than lean patients, allowing a greater tolerance for stress. Moreover, the racial difference might be another crucial reason of different results in this study compared with those in previous studies [10,19]. Several studies [26–28] demonstrated that there were significant racial differences in clinical outcomes and responsiveness to warfarin in patients with AF. Additionally, we found that renal dysfunction might be a contributor to a greater risk of cardiac or cerebral death and all-cause death in patients with AF, which has been reported by a series of studies [29,30]. In our study, renal dysfunction did not appear to be an independent risk factor for stroke and thromboembolism. Possible reasons for this lack of finding might include a lack of stratification of renal function and no sex stratification test [31].

## Limitations

This study was limited by the relatively small number of patients, which represents a single-center experience. Therefore, our results may not be generalizable to all AF populations in China. We did not differentiate subtypes of AF, but current strategies for consideration of oral anticoagulation do not distinguish AF subtypes [32]. BMI is not the only tool for measuring obesity. Other indices, such as the waist-to-hip ratio or weight-to-height ratio, were not recorded. In the present study, there were insufficient data on patients with a BMI≥30.0 kg/m^2^ and <18.5 kg/m^2^ because there were only 92 and 44 patients, respectively. Therefore, we had insufficient statistical power to clarify if patients with a BMI category ≥30.0 kg/m^2^ and <18.5 kg/m^2^ might contribute to the risk of stroke and thromboembolism in patients with AF. This lack of data could also be why we did not find a significant increase in ischemic stroke in the BMI category ≥30.0 kg/m^2^. An investigation in a much larger AF cohort is necessary. Furthermore, perhaps more important, the racial differences and the lack use of warfarin in the study population might affect the incidences of cardiovascular events and impacts of BMI on risks of clinical outcomes. In our cohort, only 21.70% of patients were female, which could affect the sex-specific risk for adverse events [33,34]. Changes in BMI may have occurred during follow-up, and this was not taken into account. However, BMI tends to be fairly stable over time, especially in older adults [35]. In a cohort study, it is difficult to deal with internal controls consisting of groups that are coarsely inhomogeneous. Therefore, a case-control study could provide a higher level of evidence.

## Conclusion

BMI might affect the prognostic outcomes of AF patients. Overweight and obesity may be contributors to a greater risk of ischemic stroke and thromboembolism in Chinese patients with AF, and are associated with a significantly lower risk of all-cause mortality compared with normal weight. Underweight might be an independent predictor for all-cause mortality.
